# Inhibition of zymosan-induced kidney dysfunction by tyrphostin AG-490

**DOI:** 10.1186/1476-9255-6-13

**Published:** 2009-05-05

**Authors:** Petya Dimitrova, Valeriya Gyurkovska, Irina Shalova, Luciano Saso, Nina Ivanovska

**Affiliations:** 1 Department of Immunology, Institute of Microbiology, Bulgarian Academy of Sciences, Sofia, Bulgaria; 2 School of Bioengineering and Bioinformatics, Lomonosov Moscow State University, Moscow, Russia; 3 Department of Physiology and Pharmacology "Vittorio Erspamer", Sapienza University of Rome, Italy

## Abstract

**Background:**

Zymosan-induced shock has been associated with an increased production of pro-inflammatory cytokines and mediators, causing a generalized dysfunction of liver, lung and kidneys. Herein, we investigate the effects of tyrphostin AG-490 on the early inflammation and on the late renal injury provoked by zymosan injection.

**Methods:**

Shock was induced by intraperitoneal injection of zymosan in a dose of 0.8–1.0 mg/g body weight in BALB/c mice and 0.8 mg/g body weight in SCID mice. Tyrphostin AG-490 was administered intraperitoneally in a dose of 5 mg/kg immediately after shock induction. Blood, peritoneal lavage and kidneys were collected at certain time points after zymosan injection. The levels of MIP-1α, RANTES, IL-6, IL-10, α1-antitrypsin and C5a in plasma were determined by ELISA. The number of IL-10-secreting cells in peritoneum was assayed by ELISPOT. Kidney function was monitored by measurement of urine/plasma creatinine levels and proteinuria. Histological assessment of renal injury was performed in a blinded fashion after hematoxylin/eosin staining. Immunohistochemistry analyses were used to evaluate the expression of C5aR, STAT1, STAT3 and the binding ability of IgGs in kidneys.

**Results:**

Tyrphostin AG-490 attenuated the early phase of zymosan-induced shock via inhibition of MIP-1α, RANTES and C5a plasma levels and via elevation of IL-10 in plasma. The drug increased IL-10 production in peritoneum and the number of IL-10-secreting peritoneal cells. AG-490 was able to retain the time of coagulation and the level of α1-antitrypsin to normal values. At the late stage of shock, AG-490 decreased scores of tubular injury, cell infiltration and glomerular lesions in parallel with diminished creatinine plasma level and protein excretion. These beneficial effects of AG-490 were related to lowered levels of circulating IL-6, MIP-1α and C5a, and to inhibited expression of STAT1, STAT3 and C5aR in kidneys. The drug diminished the production of zymosan-specific IgG antibodies and hindered the glomeruli from IgGs recognition.

**Conclusion:**

Tyrphostin AG-490 reduced the magnitude of the initial inflammatory response in zymosan-induced shock and prevented the development of severe kidney dysfunction. Our data suggest that the drug might be used as a therapeutic approach in cases where shock is combined with acute renal injury.

## Background

Septic shock is a complex inflammatory disease associated with a high rate of mortality. It starts with an overwhelmed immune response to infectious agents or their products in which the activated macrophages, neutrophils and the complement system play important roles. Cytokines and inflammatory mediators produced and secreted at first hours can induce organ failure and damage. Kidney involvement has been often observed in septic shock patients [1] and contributed to high mortality rate [2,3]. While high levels of the pro-inflammatory TNF-α and IL-6 favor the renal injury [4], IL-10 has a suppressive effect and attenuates the kidney inflammation [5]. In the model of zymosan-induced shock, kidney dysfunction is mainly evaluated by measurement of organ size and of serum creatinine level [6]. Recent report has shown an up-regulated expression of IL-6, TNF-α and IL-1β mRNA in kidneys during the middle phase of zymosan-induced shock [7]. In this study, strongly elevated level of IL-10 mRNA determines the enhanced resistance of kidneys to zymosan-induced inflammation. The renal tubular necrosis has been observed at the late stage of the disease [8], but more investigations are required to fully describe the kidney involvement in this animal model.

Zymosan is recognized by immune cells through Toll-like receptors 2 and 6 (TLR2, TLR6) that trigger the MyD88-mediated NF-kB activation and cytokine production [9,10]. The binding of zymosan to the C-type lectin receptors such as dectin-1 receptor induces phagocytocis [11]. Besides immune cells, zymosan can activate directly the alternative complement pathway resulting in extensive C5a generation [12]. Previously, we have observed that lowered C5a levels in peritoneum and in circulation of properdin-deficient mice improved the course of zymosan-induced inflammation [13]. C5a increases neutrophil chemotaxis and the production of superoxide ions, vasodilation and apoptosis [14]. It has also been implicated in the pathology of human and animal renal diseases [15] and recently, some therapeutic strategies are pointed on the inhibition of C5a or its receptor [16].

Tyrphostin AG-490 is a JAK2 kinase inhibitor that targets the cytokine-dependent STAT signalling pathway. The drug has a beneficial effect in a model of autoimmune encephalomyelitis [17] and inhibits the abnormal cell proliferation in patients with lymphoblastic leukemia, acute myeloid leukemia and Sezary syndrome [18,19]. The restriction of JAK/STAT pathway in macrophages by AG-490 diminishes IFN-γ-induced nitric oxide synthase expression and nitric oxide secretion, and inhibits TNF-α production triggered by high mobility group box 1 protein (HMGB1) [20,21]. In respect to kidneys, AG-490 attenuates experimental nephritic syndrome and ischemia/reperfusion kidney injury [22,23]. According to our previous investigations AG-490 inhibited TLR4- and TLR9-induced IL-12 and nitric oxide production by peritoneal macrophages and attenuated the liver abnormalities in aseptic shock [24,25]. Herein, we have extended our investigations and we have evaluated the effects of AG-490 on zymosan-induced kidney dysfunction.

## Methods

### Reagents

Zymosan A from *Saccharomyces cerevisiae *(Sigma-Aldrich, Munich, Germany) was suspended in endotoxin-free water at a concentration of 40 mg/ml, homogenized by sonic emulsification, autoclaved for 30 min and stored in aliquots at -20°C. Tyrphostin AG-490 (2-Cyano-3-(3,4-dihydroxyphenyl)-N-(benzyl)-2-propenamide) was obtained from Sigma-Aldrich (Munich, Germany) and was dissolved to 5 mg/ml in 75% ethanol, and frozen at -20°C.

### Animals

Female BALB/c or SCID mice, 8–10 weeks old weighing 20–25 g, were purchased from The Charles River Laboratories (Wilmington, Massachussets, USA). They were maintained in specific pathogen free environment and had free access to water and standard food. All experiments were conducted in accordance with The National Guideline for the Care and Use of Laboratory Animals (Decree No 14/19.07.2000) and were approved by the Animal Care Committee at the Institute of Microbiology, Sofia.

### Experimental design

Shock was induced by intraperitoneal injection of 1 mg/g body weight of zymosan (0.5 ml) in BALB/c mice (n = 15/group/experiment). In our experiments we have used female mice. Any differences in the development of disease between male and female animals have not been reported. The stock solution of tyrphostin AG-490 was diluted to 1 mg/ml in endotoxin-free phosphate-buffered saline (PBS; Cambrex Bioscience, Verviers, Belgium) and was administered intraperitoneally in a dose of 5 mg/kg. The animals were treated with AG-490 immediately after the induction of shock. Control groups received vehicle solution (0.5 ml) containing 1.2% ethanol in endotoxin-free PBS or 5 mg/kg AG-490. The survival of mice was monitored for 21 days. Blood, peritoneal lavage and kidneys were collected at certain time points after shock induction. In another set of experiments, shock was induced in mice with severe combined immunodeficiency (SCID). SCID mice were injected with zymosan in a dose of 0.8 mg/g body weight because in our previous study the injection of 1 mg/g body weight of zymosan caused 100% mortality within 24 h [26]. BALB/c mice were injected with the same dose of zymosan. AG-490 was administered in a dose of 5 mg/kg (n = 15/group/experiment). Blood and kidneys were collected on day 21 of shock.

### Preparation of peritoneal lavage and isolation of peritoneal cells

Peritoneal lavage was obtained 4 h after the injection of zymosan (n = 5/group) by washing the peritoneal cavity with 2 ml sterile RPMI-1640 medium (Biowhittaker™, Cambrex, Verviers, Belgium). The supernatants were collected after centrifugation at 1200 × g for 10 min and immediately frozen at -70°C. The cell pellets were resuspended in RPMI-1640 medium containing 5% fetal calf serum (FCS) and were dispensed in glass Petri dishes (5 ml/dish). After incubation for 1 h at 37°C, 5% CO_2_, the non-adherent cells were carefully aspirated, washed with PBS and counted. The adherent cells were gently detached by scraping with a silicone rubber, washed and counted. Both populations of peritoneal cells were resuspended in 10% FCS/RPMI-1640 at a concentration of 1.0 × 10^6 ^cells/ml and were used in ELISPOT assay for determination of IL-10-secreting cells.

### Measurement of cytokines and chemokines

Blood was collected in heparin-containing glass tubes by retro-orbital puncture (n = 5/group). Plasma was obtained by centrifugation at 3000 × g for 10 min at 4°C, and was frozen at -70°C. The levels of IL-10, IL-6, MIP-1α and RANTES were measured in plasma and in peritoneal lavage by ELISA. The quantitative ELISA kits (PeproTech EC Ltd, London, UK) were with detection limits 47 pg/ml for IL-10, 62 pg/ml for IL-6, 8 pg/ml for MIP-1α and 16 pg/ml for RANTES, respectively.

### Detection of IL-10-secreting peritoneal cells

The number of IL-10-secreting peritoneal cells was determined by ELISPOT assay. Nitrocellulose-backed 96-well microtiter plate (Millipore, Billerica, Massachussets, USA) was coated with purified rabbit anti-mIL-10 antibody (10 μg/ml, PeproTech, London, UK). The plate was incubated overnight at 4°C and then washed three times with PBS. Non-adherent and adherent peritoneal cells (1.0 × 10^5 ^cells/well), and a positive control of recombinant IL-10 (250 μg/ml; 100 μl/well; PeproTech, London, UK) were added at triplicates to the plate and were stimulated with zymosan (20 μg/ml) for 18 h at 37°C. The cells were then removed by washing with PBS and the unspecific binding was blocked with 5% bovine serum albumin (BSA)/PBS for 1 h at room temperature. The biotinylated rabbit anti-mIL-10 antibody (0.250 μg/ml; 100 μl/well; PeproTech, London, UK) was added for 2 h at room temperature. The plate was washed and incubated with avidin peroxidase conjugate (1:1000 diluted; 100 μl/well; PeproTech, London, UK) for 30 min. The substrate solution containing 3-amino-9-ethylcarbazole (200 μl/well; AEC; Sigma-Aldrich, Munich, Germany) was used. The color reaction was stopped with dH_2_O and air-dried overnight before spot enumeration using a light microscope (Boeco, Hamburg, Germany) at 1 × 100 magnification. The results were expressed as counted spots per 1.0 × 10^5 ^cells.

### Detection of zymosan-specific IgG antibodies

The serum level of zymosan-specific IgG antibodies was determined by ELISA as described [27]. ELISA 96 well test plates (Greiner Bio-One GmbH, Essen, Germany) were coated with zymosan (100 μg/well) in PBS, blocked with 2% BSA/PBS and incubated with serum samples (1:100 diluted) for 2 h at room temperature. The secondary peroxidase-conjugated anti-mouse IgG antibody (1:10 000 diluted; Sigma-Aldrich, Munich, Germany) was incubated for 1 h at room temperature. The substrate *o*-phenylenediamine (Sigma-Aldrich, Munich, Germany) was used to develop the colorimetric reaction. The absorbance was measured at 492 nm in a microplate reader (BioTek Instruments Inc, Winooski, Vermont, USA). The data were obtained using the Gen 5.0 software (BioTek Instruments Inc). Samples were measured in triplicates and their absorption was normalized to that of the control positive serum. The results were expressed in relative units (RU) ± standard deviation.

### Measurement of plasma C5a

ELISA 96-well plates (Greiner Bio-One GmbH, Essen, Germany) were coated with rat anti-mouse C5a antibodies (BD Biosciences, Erembodegem, Belgium) overnight and blocked with 2% BSA/PBS for 1 h at room temperature. Plasma samples (diluted 1:5) and serial dilutions of C5a (BD Biosciences, Erembodegem, Belgium) were added in triplicates and incubated for 2 h at room temperature. After washing, biotinylated rat anti-mouse C5a antibodies (BD Biosciences, Erembodegem, Belgium) was added and detected with avidin-peroxidase (1:1000, PeproTech EC Ltd, London, UK). The results were calculated from a standard curve plotting the absorbance values against the concentrations of C5a and were expressed in picograms per ml.

### Coagulation time and plasma level of α1-antitrypsin

The level of α1-antitrypsin and the coagulation time were determined as previously described [13,28].

### Functional assessment of renal injury

Blood was collected at certain time points of zymosan injection (n = 5/group). The plasma and urine creatinine levels (milligrams per deciliter) were determined by alkaline picric acid method using a standard laboratory kit (Dialab, Wiener Neudorf, Austria). The protein level in urine was measured by Bradford assay. The protein excretion showing the glomerular filtration rate was expressed as milligrams urinary proteins per milligrams urinary creatinine.

### Histolopathological assessment of renal injury

Kidneys were fixed in 10% paraformaldehyde/PBS (pH 7.4). The organs were embedded in paraffin and sections with thickness 4 μm were cut by rotary microtome (Accu-Cut^® ^SRM™ Sacura Finetek, Tokyo, Japan). The slides were stained with hematoxylin and eosin (H&E) and were examined with a light microscope (BM-180 T/PL, Boeco, Hamburg, Germany) using a 1 × 100 or 1 × 400 lens. Images were captured with a coupled device camera and exported to Adobe Photoshop 7.0 (Adobe Systems, Munich, Germany).

All histological assessments were performed in a blinded protocol. The degree of renal injury was graded semi-quantitatively in at least 30 cross-sections per mice according following characteristics: glomerular lesions, tubular vacuolization, tubular dilation, tubular necrosis and leukocyte infiltration. The 5 score system was used: score 1 = no abnormality, 2 = 10% injury; 3 = 25% injury; 4 = 50% injury; 5 = > 75% injury. The renal injury score was calculated as an average score of the mean score for each characteristic.

### Immunohistochemistry

The expression of STAT1 and STAT3 in kidneys was evaluated as previously described [25]. Kidney sections (4 μm) were immersed in 3% H_2_O_2_/60% methanol for 10 min to block endogenous peroxidase. After blocking of unspecific binding with 5% BSA/PBS, the sections were incubated for 2 h with antibodies against STAT3 (1:100 diluted, Santa-Cruz Biotechnology, Heidelberg, Germany) and STAT1 (1:500 diluted, Santa-Cruz Biotechnology, Heidelberg, Germany) or with isotype antibodies. The sections were washed with PBS, incubated with HRP-labelled anti-rabbit IgG antibody (1:2000 diluted, Sigma-Aldrich, Munich, Germany) for 30 min at room temperature and stained with DAB (3,3'-diaminobenzidine-tetrahydrochloride) substrate solution (Sigma-Aldrich, Munich, Germany) for 1 min. Kidney sections were counterstained for 30 sec with Gill's hematoxylin and studied microscopically.

To determine the renal expression of C5aR, kidney sections were permeabilized with 0.1% Triton X-100/PBS for 20 min and blocked with 5% BSA/PBS for 1 h at room temperature. After washing, the sections were incubated for 2 h at room temperature with antibody against C5a receptor (0.2 mg/ml; 1:200 diluted; BD Biosciences, Erembodegem, Belgium). Isotype antibody (rabbit anti-mouse IgG; Sigma-Aldrich, Munich, Germany) was used as a background staining control. The secondary FITC-labelled anti-rabbit IgG antibody (1:120 diluted; Sigma-Aldrich, Munich Germany) was added for 40 min. The sections were washed and examined with a fluorescent microscope (BM-180 T/PL, Boeco, Hamburg, Germany).

The glomerular binding of IgG antibodies was evaluated in kidney sections after 40 min incubation with 1:100 diluted sera pooled from healthy mice (normal serum) or from zymosan-immunized mice (ZY-positive serum). The latter was obtained on day 21 post-zymosan injection and contained high titer of anti-zymosan IgG antibodies. Secondary FITC-conjugated anti-mouse IgG (Fc specific) antibody (1:500 diluted, Sigma-Aldrich, Munich, Germany) was added for 15 min and the binding of IgG antibodies to gromeruli was examined with a fluorescent microscope.

### Statistical analyses

Data are expressed as mean ± SD. Statistical significance of differences in survival rate was analyzed by two-way ANOVA test. For paired data Student's *t *test was used. Differences were considered significant when p < 0.05. Statistical analysis was accomplished using InStat3.0 and GraphicPad Prism 5.0 (GraphPad Software Inc, La Jolla, California, USA).

## Results

### Effect of AG-490 on the survival rate, coagulation time and C5a, α1-antitrypsin levels

**Figure 1:**
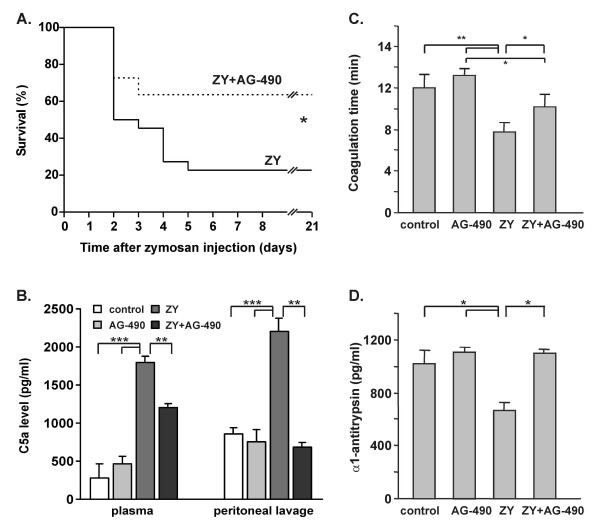
**Effect of AG-490 on zymosan-induced inflammation**. A, The administration of AG-490 (5 mg/kg) increased the survival rate of mice injected with 1 mg/g body weight of zymosan (n = 15/group). B, AG-490 inhibited the level of C5a in peritoneal lavage and in plasma 4 h after zymosan injection. C-D, AG-490 rendered the time of coagulation and the plasma level of α1-antitrypsin to normal range 4 h after the induction of shock. The data represent mean ± SD from 3 independent experiments with 5 mice/group; *p < 0.05, **p < 0.01 and ***p < 0.001; Control groups of healthy mice were treated with vehicle (control) or AG-490 (AG-490), shock mice were treated with vehicle (ZY) or with AG-490 (ZY+AG-490); ANOVA test was used to analyze the survival data and Student's *t *test to compare other parameters.

In our previous experiments, shock mice were treated with AG-490 in doses ranging from 1 to 10 mg/kg and in different schedules [25]. The dose of 5 mg/kg administered immediately after the injection of zymosan (1 mg/g body weight) was determined as the most effective in inhibiting the mortality and was used herein. AG-490 significantly increased the survival rate of BALB/c mice (Fig. 1A). Four hours after shock induction, the elevated C5a production was strongly inhibited in AG-490-treated mice (Figure 1B). The drug administered in healthy mice did not influence the plasma and peritoneal level of C5a (Figure 1B). Zymosan-injected mice showed a reduced coagulation time (Figure 1C) and a reduced serum level of α1-antitrypsin (Figure 1D). The administration of AG-490 rendered both parameters to normal values (Figure 1C, D). AG-490 slightly increased the coagulation time in healthy mice without having an effect on α1-antitrypsin level (Figure 1C, D).

### Tyrphostin AG-490 inhibits the levels of MIP-1α and RANTES and favors IL-10 production in peritoneum and plasma

Zymosan injection (1 mg/g body weight) elevated the level of MIP-1α and RANTES and increased IL-10 production in plasma and in peritoneal lavage at 4 h (Table 1). The administration of AG-490 significantly diminished the levels of MIP-1α and RANTES and enhanced additionally the production of IL-10 in mice with shock (Table 1). The substance itself did not markedly change the levels of the three mediators neither in the peritoneum nor in the circulation of healthy mice and slightly elevated the number of peritoneal cells (1.32 ± 0.15 × 10^6 ^cells/ml in control group versus 1.60 ± 0.09 × 10^6 ^cells/ml; p > 0.05).

**Table 1:** Effect of AG-490 on the MIP-1α, RANTES and IL-10 levels in peritoneum and plasma

Groups^a^	MIP-1α (pg/ml)		RANTES (pg/ml)		IL-10 (pg/ml)	
	peritoneum	plasma	peritoneum	plasma	peritoneum	plasma
Control	120 ± 28	140 ± 28	145 ± 18	80 ± 20	20 ± 2.0	30 ± 1.8
AG-490	120 ± 30	116 ± 15	125 ± 20	65 ± 15	25 ± 2.0	28 ± 2.0
ZY	1200 ± 58	800 ± 48	580 ± 40	220 ± 35	780 ± 88	420 ± 45
ZY+AG-490	280 ± 36*	380 ± 36*	360 ± 50*	160 ± 30	920 ± 86*	640 ± 54*

^a^The peritoneum and plasma level of mediators was determined in healthy mice treated with vehicle (Control) or AG-490 (AG-490) and in shock mice treated with vehicle (ZY) or AG-490 (ZY+AG-490). The plasma and peritoneal fluid were collected 4 h after zymosan injection. Data are expressed as mean ± SD (n = 5/group) and were compared by Student's *t *test. *p < 0.01 versus zymosan-injected group.

### Tyrphostin AG-490 increases the number of IL-10 secreting cells in peritoneum

In order to determine which population is responsible for the enhanced IL-10 secretion in peritoneum, the cells were separated into two populations: non-adherent and adherent peritoneal cells (Table 2). In healthy mice we were not able to detect IL-10-producing cells even after zymosan stimulation in vitro. IL-10-secreting cells were found in non-adherent cell population 4 h after zymosan injection, but more IL-10 producers appeared in adherent cell population. The number of IL-10-secreting adherent peritoneal cells increased after zymosan restimulation in vitro. The administration of AG-490 enhanced the number of IL-10 producing cells in non-adherent and in adherent peritoneal populations. In AG-490-treated mice, the zymosan restimulation of peritoneal cells did not markedly change the number of IL-10 producers.

**Table 2:** Effect of AG-490 on the number of IL-10-producing cells in peritoneum

Cell populations^a^	Treatment^b^	IL-10 producing cells (spots/1.0 × 10^5^cells)^c^	
		unstimulated	ZY-stimulated
Non-adherent peritoneal cells	Control	N.D.	N.D.
	ZY	4.0 ± 1.0	6.0 ± 2.8
	ZY + AG-490	9.0 ± 3.1*	10.0 ± 5.5
Adherent peritoneal cells	Control	N.D.	N.D.
	ZY	12.7 ± 6.8	27.0 ± 8.9
	ZY + AG-490	20.5 ± 4.5**	27.2 ± 7.2

^a^Peritoneal cells were isolated 4 h after shock induction and were separated in non-adherent and adherent cell populations.

^b^ELISPOT assay was used to determine the number of IL-10-producing cells in peritoneal lavage from healthy mice (Control), untreated (ZY) or treated with AG-490 (ZY+AG-490) mice with shock. N.D denotes not detectable.

^c^Cell populations were cultured for 18 h in the absence (unstimulated) or the presence (ZY-stimulated) of zymosan.

Data are expressed as mean ± SD (n = 5/group) and were compared by Student's *t *test.

**p < 0.01 and *p < 0.05 versus zymosan-injected group.

### Tyrphostin AG-490 inhibits the zymosan-induced kidney dysfunction

**Figure 2:**
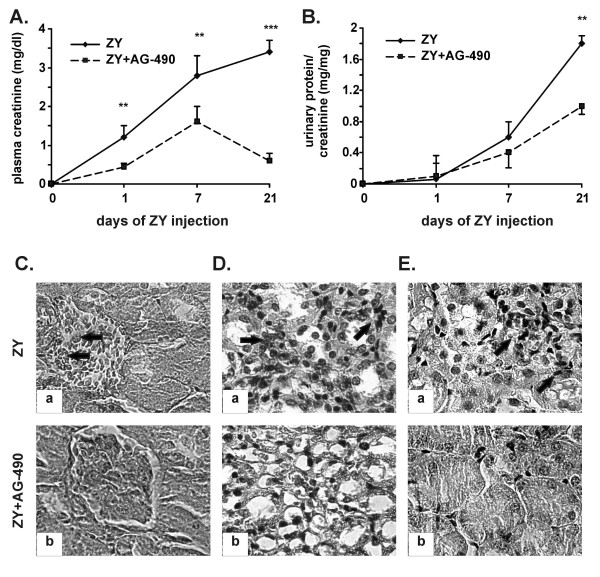
**Effect of AG-490 on kidney dysfunction induced by zymosan**. A, The drug inhibited the plasma level of creatinine. B, AG-490 prevented the development of proteinuria. The data represent the mean ± SD from 3 independent experiments including 5 mice/group; **p < 0.01 and ***p < 0.001 versus zymosan-injected group; Student's *t *test. C-E, Histological evaluation of kidneys. The representative data from 2 experiments (n = 5/group) showed glomerular lesions (indicated with arrows on Ca; 1 × 400), massive cell infiltration in renal medulla (indicated with arrows on Da, 1 × 400) and tubular necrosis (indicated with arrows on Ea; 1 × 400) in shock mice (ZY) on day 21 of zymosan injection. The described pathology was not found in AG-490-treated mice (Cb, Db, Eb; ZY+AG-490).

The plasma level of creatinine started to increase after day 1 of zymosan injection. The highest levels of creatinine were detected in plasma on day 21 and were completely abolished in AG-490-treated mice (Figure 2A). The proteinuria developed progressively from day 7 to day 21 of shock induction and was reduced in AG-490-treated mice (Figure 2B). The glomerular lesions (Figure 2Ca, see arrows), the massive cell infiltration in renal medulla (Figure 2Da, see arrows), the intensive tubular necrosis (Figure 2Ea, see arrows) were seen in kidneys of zymosan-injected mice. Histopathological analysis showed the loss of glomerular structure in 10% of injured kidneys (score 2.1 ± 0.1; Table 3) and the cell influx in medulla and cortex of more than 75% of kidneys (score 5.0 ± 0.2; Table 3). The administration of AG-490 significantly reduced the score of renal injury (Table 3). Glomerular lesions were nearly absent in AG-490-treated mice (Figure 2Cb, score 0.50 ± 0.05; Table 3). The cell infiltration and tubular injury were less severe in result of AG-490 administration (Figure 2Db, Eb; Table 3).

**Table 3:** Histology examination of kidney damage

Characteristics^a^	Groups	
	ZY	ZY+AG-490
Glomerular lesions	2.10 ± 0.10	0.50 ± 0.05**
Tubular vacuolization	4.90 ± 0.15	3.60 ± 0.10*
Tubular dilation	2.40 ± 0.10	1.50 ± 0.09*
Tubular necrosis	1.50 ± 0.05	0.50 ± 0.09*
Leukocyte infiltration	5.00 ± 0.20	2.95 ± 0.15**
Renal injury score^b^	15.00 ± 2.20	9.05 ± 2.15**

^a^Kidneys were collected on day 21 of zymosan injection from untreated (ZY) or treated with AG-490 (ZY+AG-490) mice.

^b^The renal injury score was calculated as an average score of the mean score for each characteristic. Data are calculated according to the examination of 30 sections/mice (n = 5/group) in 2 experiments. The results are expressed as mean ± SD and are compared by Student's t test. **p < 0.01 and *p < 0.05 versus zymosan-injected group.

### Tyrphostin AG-490 inhibits the plasma levels of IL-6 and MIP-1α and the renal expression of STAT1 and STAT3

**Figure 3:**
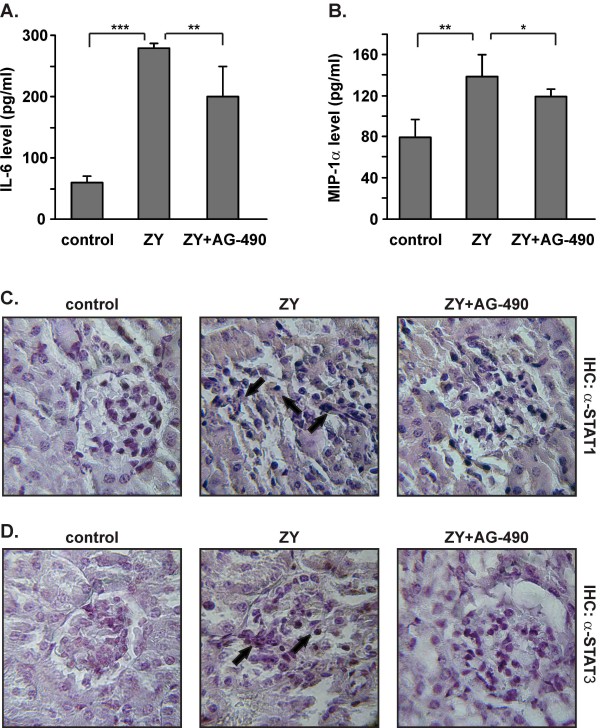
**Effect of AG-490 on the production of IL-6 and MIP-1α in plasma and on the renal expression of STAT1 and STAT3**. A-B, AG-490 diminished the plasma levels of IL-6 and MIP-1α on day 21 of zymosan injection. The data represent the mean ± SD from 3 independent experiments (5 mice/group); *p < 0.05 and **p < 0.01 versus zymosan-injected group; Student's *t *test. C-D, The representative data from 1 experiment (n = 5/group) showed increased STAT1 (indicated with arrows on C; 1 × 400) and STAT3 expression in kidneys (indicated with arrows on D; 1 × 400) on day 21 of zymosan injection (ZY group). The administration of AG-490 decreased the renal expression of both transcription factors (C-D; ZY+AG-490).

At late stage of shock, AG-490 diminished the plasma levels of IL-6 (Figure 3A) and MIP-1α (Figure 3B). No changes were noticed in healthy mice injected with AG-490 (data not shown). Since circulating cytokines can trigger JAK/STAT pathways in kidneys, we evaluated the renal expression of STAT3 and STAT1 molecules. Immunohistological analyses showed the positive staining with anti-STAT1 antibodies of kidneys from shock mice (Figure 3C). STAT3 was detected in shock kidneys at low levels and predominantly in infiltrating cells (Figure 3D). AG-490 decreased the zymosan-induced expression of STAT1 and completely inhibited the STAT3 levels in kidneys (Figure 3C, D).

### Tyrphostin AG-490 attenuates the renal injury in SCID mice

**Figure 4:**
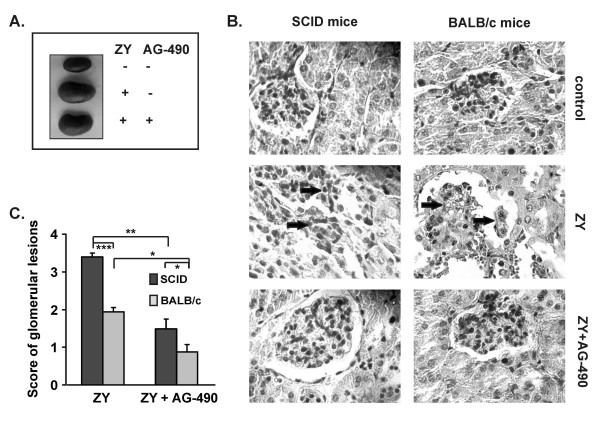
**Influence of AG-490 on zymosan-induced kidney damage in SCID and in BALB/c mice**. A, B, The administration of AG-490 reduced the kidney enlargement and prevented the abnormalities in glomerular structure in SCID and BALB/c mice on day 21 post-zymosan injection. C, The score of glomerular lesions was higher in SCID mice than in BALB/c mice. Data were expressed as mean ± SD from 2 independent experiments with 5 mice/group *p < 0.05, **p < 0.01 versus mice with shock; Student's *t *test.

In order to evaluate the role of acquired immunity for zymosan-induced renal injury, SCID mice (without functional B and T cells) and BALB/c mice were injected with 0.8 mg/g body weight of zymosan. On day 21 SCID mice showed significantly increased kidney size, compared to the control group of healthy animals (Figure 4A). AG-490 diminished the kidney enlargement in 70% of shock mice and prevented the loss of glomerular structure induced by zymosan (Figure 4B). Notably, the glomerular lesions were exhibited more often in SCID mice than in BALB/c mice (score of glomerular lesions 3.4 ± 0.1 in SCID mice versus score 1.5 ± 0.2 in BALB/c mice; Figure 4C).

### Tyrphostin AG-490 inhibits plasma C5a level and C5aR expression in kidneys

**Figure 5:**
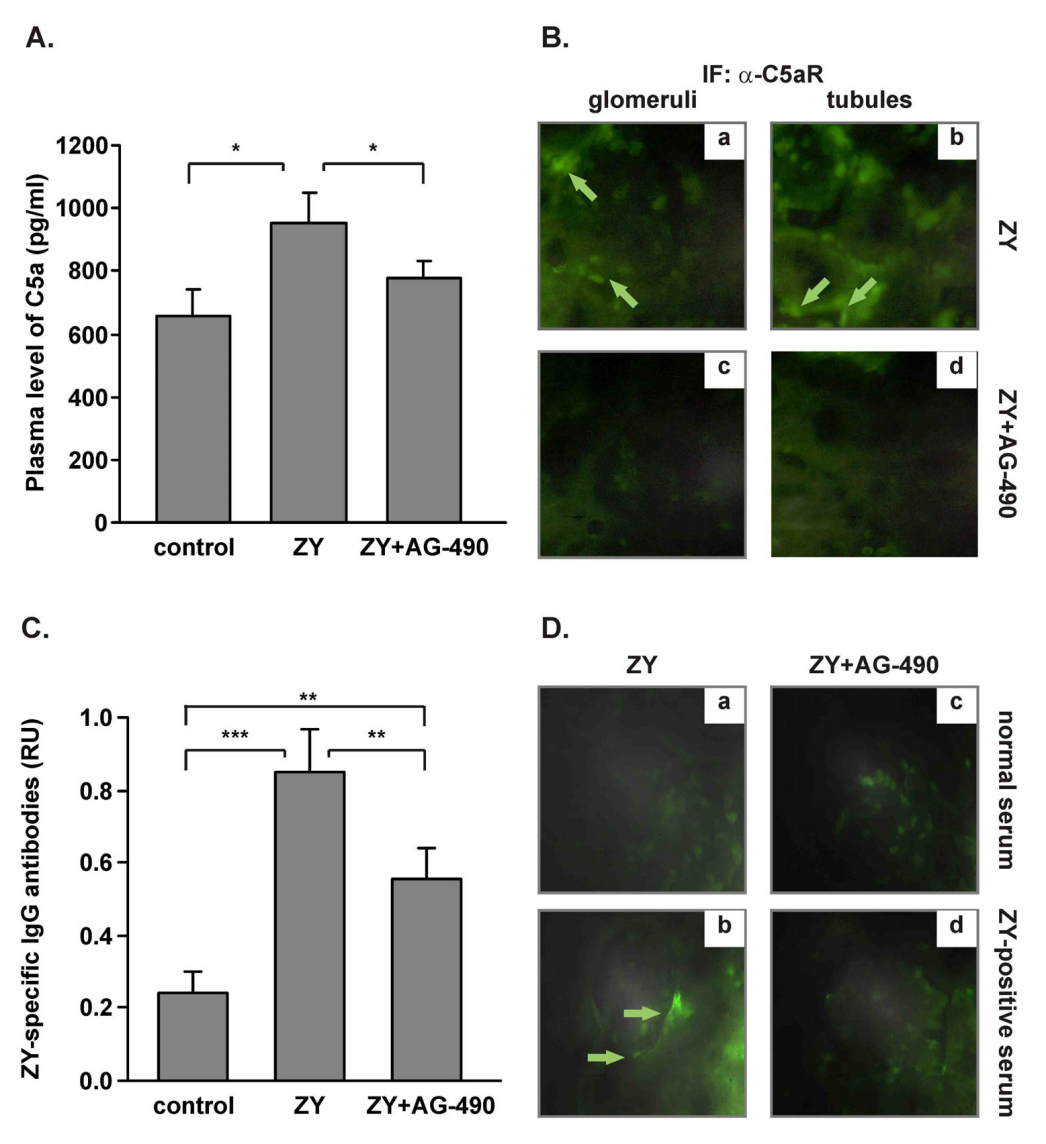
**Effect of AG-490 on plasma C5a and zymosan-specific IgG antibody levels, on C5aR expression in kidneys and on the IgGs binding to glomeruli**. A, AG-490 diminished the level of circulating C5a on day 21 post-zymosan injection. AG-490 inhibited C5aR expression in kidneys. Infiltrating cells in glomeruli, Ba and tubular epithelial cells, Bb of shock kidneys were positive for C5aR staining (arrows). Bc, d, The renal C5aR expression was undetectable in AG-490-treated mice; 1 × 400; *p < 0.05 versus mice with shock, Student's *t *test. C, Zymosan injection induced anti-zymosan IgG antibodies production that was significantly inhibited by AG-490; **p < 0.01 versus zymosan injected group. The results are expressed as mean ± SD of 5 mice per group from 2 experiments. D, The representative data from 2 experiments showed the glomerular binding of IgGs isolated from zymosan-immunized mice (b, d, ZY-positive serum) but not of IgGs isolated from healthy mice (a, c; normal serum). Arrows showed the areas of positive staining.

On day 21 AG-490 significantly inhibited the high amount of C5a in circulation of shock mice (Figure 5A). In the group of AG-490-treated healthy mice the level of C5a was similar to the untreated healthy controls (data not shown). Immunohistochemical analysis of kidney sections from mice with shock revealed positive staining for C5a receptor in glomeruli (Figure 5Ba) and in tubular epithelial cells (Figure 5Bb). In glomeruli, C5aR expression was detected on infiltrating cells (see arrows). The C5aR expression was nearly undetectable in kidneys of AG-490-treated mice (Figure 5Bc, d).

### Tyrphostin AG-490 inhibits the level of zymosan-specific IgG antibodies and affects IgGs binding to renal glomeruli

The circulating zymosan-specific IgG antibodies were determined in mice with zymosan-induced shock on day 21 that was significantly inhibited in AG-490-treated mice (Figure 5C). In respect to kidneys, immunohistochemistry analyses in shock mice revealed the recognition of the glomerular structure by IgGs pooled from zymosan-immunized mice (ZY-positive serum; Figure 5Db). In control sections, IgGs from healthy mice (normal serum; Figure 5Dd) did not bind to glomeruli. In AG-490-treated mice, neither normal nor ZY-positive sera bound to glomeruli (Figure 5Da, c). After examination of 30 glomeruli/mice, the positive staining was observed in 1 of 5 mice from AG-490-treated group.

## Discussion

In the present study we have estimated the effect of JAK2 inhibitor tyrphostin AG-490 on zymosan-induced inflammation with focus on kidney dysfunction. Tyrphostin AG-490 increased the survival and attenuated the initial phase of shock by inhibition of MIP-1α and RANTES production. AG-490 enhanced IL-10 levels and the numbers of IL-10 producing peritoneal cells in shock mice. IL-10-positive spots were found in non-adherent and in adherent peritoneal cell populations. In non-adherent population, B-1 cells are most likely the source of IL-10. These cells constitute 10–15% of the total peritoneal cell pool, express high levels of surface IgM and downregulate macrophage effector functions such as phagocytosis and cytokine production [29]. In our study, the adherent peritoneal cells contributed to the enhanced IL-10 levels in peritoneum. Recently, it has been shown that the depletion of resident macrophages (but not monocytes) in IL-10 deficient mice with shock resulted to an intensive accumulation of polymorphonuclear cells (PMNs) in peritoneum [30]. In AG-490-treated mice 4 h after zymosan injection we detected an increased number of IL-10-producing adherent cells along with an abolished neutrophil influx. However, it should be considered that the strongly elevated IL-10 levels are not always beneficial for the disease as they can inhibit cell-mediated immunity, can induce immunosuppression, T cell anergy and tolerance that can worsen the symptoms of disease.

Recent study provided an evidence for an interaction between complement system and TLR2 pathway [31]. In this investigation, TLR2/6 signalling leading to NF-kB activation was enhanced by the anaphylatoxin receptors C5aR and C3aR resulting in complement-dependent elevation of plasma TNF-α and IL-6 levels. In the initial phase of zymosan-induced shock we found an excessive generation of C5a in plasma and in peritoneal lavage. High levels of circulating C5a can affect the coagulation directly or indirectly via cytokines [32,33]. In AG-490-treated mice the normal coagulation time was found together with decreased C5a plasma level. The appropriate activation of pro-thrombin system in AG-490-treated mice was attended by normal levels of acute phase protein α1-antitrypsin. This protein is a regulator of coagulation that inhibits the activation of protein C and inactivates harmful extracellular elastase [33].

Increased STAT3 expression in kidneys has been detected during severe oxidative stress [34] and glomerulonephritis [35]. The contribution of STAT1, STAT4 and STAT6 for renal pathology has also been provided [36,37]. Based on these studies, the strategies limiting the activation of JAK/STAT pathway may represent a novel approach to treat renal diseases. The abnormalities in kidneys were poorly described in zymosan-induced shock and the renal dysfunction was monitored mainly by changes in creatinine level [6]. In our study, the alteration in the glomerular filtration rate was found when the plasma level of creatinine was strongly increased. The administration of AG-490 diminished the amounts of plasma creatinine and prevented further development of proteinuria. These effects of tyrphostin were accompanied with the lack of changes in the glomerular structure and with decreased leukocyte infiltration, tubular dilation and vacuolization and inhibited STAT1 and STAT3 expression. Consistent with our data are the findings in models of nephritic syndrome and of renal ischemia/reperfusion injury showing the inhibited STAT1 and STAT3 expression and phosphorylation in kidneys after AG-490 administration [22,23]. STAT1 and STAT3 are activated in renal cells or in infiltrating effectors after cytokine receptors ligation [38]. In macrophages, TLRs signalling can interfere with the JAK/STAT cytokine pathways [39]. TLR2 synergizes with IFN-γ-induced STAT1 gene expression and suppresses IL-10-induced STAT3 activation [40]. In respect to kidneys, TLR2 was found to be constitutively expressed on human and mouse kidneys and TLR2 deficiency protects from the renal ischemia-reperfusion injury [41]. However, more data showing the crosstalk between TLR2 and JAK/STAT pathways in kidneys are required.

Previously, we have observed that AG-490 substantially reduced the elevated serum levels of TNF-α induced by zymosan. Thus, tyrphostin can influence shock development directly or indirectly through TNF-α action on other cytokines [25]. We established that on day 21 of shock AG-490 inhibited the plasma level of pro-inflammatory MIP-1α and IL-6. These mediators in circulation probably can contribute to the zymosan-induced kidney dysfunction. MIP-1α binds to chemokines receptors CCL1 and CCL5 expressed on differentiated macrophages [42]. In kidneys, MIP-1α can provoke the massive accumulation of macrophages and can maintain the renal injury [43].

T cells actively participate in renal injury. They are mainly with Th1 phenotype and produce TNF-α and IFN-γ [44]. The contribution of Th1 cells in renal pathology has been well described in T bet deficient animals, which lack a transcription factor promoting Th1 cell differentiation [45]. The data about the role of B cells in kidney disease are limited. Recent studies have shown that B-cell deficiency protected the mice from ischemic injury [46] and that the number of CD19+ B cells was decreased in damaged glomeruli [44]. In order to elucidate whether the acquired immunity contributes to zymosan-induced inflammation we have used SCID mice. The lack of functional T and B lymphocytes during shock progression resulted in the increased mortality and in exacerbation of organ injury. On day 21 we detected greater kidney enlargement than in normal mice in parallel with elevated serum creatinine, tubular injury and intestitial inflammation. Importantly, glomerular lesions occurred more often in SCID mice than in BALB/c mice. We suggest that T and B cells may protect kidneys from renal injury and may play a critical role for the self-defense mechanisms in glomeruli. This hypothesis has been partially supported by recent study showing the attenuated renal inflammation after the reconstitution of CD4+ T cells [47]. However, more investigations are required to understand the role of T and B cells in zymosan-induced kidney dysfunction and in the self-defence machinery.

IL-6 causes an increased expression of C5aR in various organs, such as lung, liver, kidney and heart during CLP-induced sepsis. The inhibition of IL-6 leads to reduced expression of C5aR and increased survival [48]. IL-6 upregulates C5aR on myeloid (thymocytes, macrophages, neutrophils) and on nonmyeloid (epithelial cells, endothelial cells) cells in lung, liver and kidney [49,50]. On day 21 plasma C5a level remained higher in shock mice, although not so extremely elevated as it was at first 4 hours. The administration of AG-490 strongly reduced circulating C5a and inhibited C5aR expression on tubular epithelial cells on day 21. In AG-490-treated mice, the negative staining for C5aR in glomeruli was related to inhibited influx of cells expressing C5aR. Low level of C5aR can prevent further activation of tubular epithelial cells and can inhibit the local production of pro-inflammatory mediators IL-8, IL-6, TNF-α, MCP-1 and RANTES, the expression of adhesion molecules and the additional activation of complement cascade [48]. While neutrophils loose immediately the surface C5aR in result of C5a/C5aR internalization [51], macrophages exerted strong responses after C5aR ligation [52]. Further study should be conducted to clarify which population expressing C5aR, macrophages or neutrophils is predominantly affected by AG-490 administration.

The AG-490 treatment significantly diminished the level of anti-zymosan IgGs showing the inhibited B cell functions. The role of IgG antibodies in the pathology of glomerulonephritis has been well documented [53]. The glomerular injury is induced by the deposition of IgG antibodies, the formation of immune complexes and the activation of complement [54] or by the engagement of Fc fragments of IgGs with cells expressing Fcγ R at the inflammatory site [55]. Based on these reports, the question whether anti-zymosan IgG antibodies can trigger immune reactions in kidney became intriguing. Immunohistochemistry analyses showed that IgGs pooled from zymosan-immunized mice were able to recognize some kidney structures in shock mice, unlike IgGs from healthy mice. This binding was observed in 10% of glomeruli with detected structural abnormalities. We suggest that new immunogenic structures can be exposed in a result of kidney damage. The antigen mimicry and the epitope spreading are common phenomena for autoimmune diseases such as rheumatoid arthritis and diabetes and are usually responsible for the perpetuation of inflammatory immune responses [56]. Similar events can occur in kidneys at the late stage of shock since zymosan induced renal injury in parallel with antibody production. However, the implications of anti-zymosan IgGs in kidney dysfunction induced by zymosan remain to be elucidated.

## Conclusion

In the present study, we have observed that AG-490 inhibited zymosan-induced kidney dysfunction via decrease of creatinine level, prevention of glomerular structural damages and proteinuria. These effects were also mediated by reduced levels of pro-inflammatory MIP-1α and IL-6 and inhibited renal STAT1 and STAT3 expression. Our results provide new data on the important role of C5a/C5aR, T and B cells for the development of renal injury in zymosan-induced shock.

## Abbreviations

TNF-α: tumor necrosis factor alpha; IL: interleukin; MIP-1α: migration inflammatory protein 1 alpha; RANTES: regulated upon activation, normal T-cell expressed and secreted; ELISA: enzyme-linked immunosorbent assay; ELISPOT: enzyme-linked immunosorbent spot assay; IgG: immunoglobulin; JAK: Janus activated kinase; STAT: signal transducers and activator of transcription.

## Competing interests

The authors declare that they have no competing interests.

## Authors' contributions

PD and NI equally contribute to the conception and design of the study, to data analysis and to the manuscript writing. VG performed the histological assessment of kidneys. IS and LS contributed to the α1-antitrypsin determination and corrected the manuscript. All authors have read and approved the final version of the manuscript.
